# Investigating Indirect and Direct Reputation Formation in Asian Elephants (*Elephas maximus*)

**DOI:** 10.3389/fpsyg.2020.604372

**Published:** 2021-01-08

**Authors:** Hoi-Lam Jim, Friederike Range, Sarah Marshall-Pescini, Rachel Dale, Joshua M. Plotnik

**Affiliations:** ^1^Domestication Lab, Department of Interdisciplinary Life Sciences, Konrad Lorenz Institute of Ethology, University of Veterinary Medicine Vienna, Vienna, Austria; ^2^Department for Psychotherapy and Biopsychosocial Health, Danube University Krems, Krems, Austria; ^3^Department of Psychology, Hunter College, City University of New York, New York, NY, United States; ^4^Department of Psychology, The Graduate Center, City University of New York, New York, NY, United States

**Keywords:** eavesdropping, third-party evaluation, image scoring, social evaluation, third-party interactions, human-animal interactions, string-pulling, elephant cognition

## Abstract

Reputation is a key component in social interactions of group-living animals and appears to play a role in the establishment of cooperation. Animals can form a reputation of an individual by directly interacting with them or by observing them interact with a third party, i.e., eavesdropping. Elephants are an interesting taxon in which to investigate eavesdropping as they are highly cooperative, large-brained, long-lived terrestrial mammals with a complex social organisation. The aim of this study was to investigate whether captive Asian elephants (*Elephas maximus*) could form reputations of humans through indirect and/or direct experience in two different paradigms: (1) a cooperative string-pulling task and (2) a scenario requiring begging. Fourteen captive Asian elephants in Thailand participated in an experimental procedure that consisted of three parts: baseline, observation, and testing. In the observation phase, the subject saw a conspecific interact with two people—one cooperative/generous and one non-cooperative/selfish. The observer could then choose which person to approach in the test phase. The elephants were tested in a second session 2–5 days later. We found no support for the hypothesis that elephants can form reputations of humans through indirect or direct experience, but these results may be due to challenges with experimental design rather than a lack of capacity. We discuss how the results may be due to a potential lack of ecological validity in this study and the difficulty of assessing motivation and attentiveness in elephants. Furthermore, we highlight the importance of designing future experiments that account for the elephants' use of multimodal sensory information in their decision-making.

## Introduction

Cooperation is defined as two or more individuals working together to obtain a mutual benefit and is frequently observed in group-living animals. For example, female elephants collectively take care of younger individuals in the herd (i.e., allomothering—Lee, 1987), wolves (*Canis lupus*) work together to hunt large prey (MacNulty et al., 2014), and male chimpanzees (*Pan troglodytes*) defend their territory together (Boesch and Boesch, 1989). The evolutionary mechanisms that underlie the expression of cooperation in social animals are well-understood (West et al., 2007), but how cooperation is maintained within a social group to increase an animal's chance of survival is not.

Reputation refers to knowing how an individual behaves in a typical situation based on what is known about that individual's behaviour in the past (Russell et al., 2008). It is another component in the social interactions of group-living animals and appears to play a key role in the establishment of cooperation. For example, when an individual has a reputation for “cooperation,” they may have more opportunities to acquire desirable resources and partners than an individual known to be “non-cooperative,” who may instead be excluded from interactions with others (Wu et al., 2016). Thus, reputation can also contribute to survival (Abdai and Miklósi, 2016).

Reputation can be formed through direct interactions between individuals or via observations of interactions by a third party, known as eavesdropping (Bonnie and Earley, 2007). Eavesdropping may be more cognitively demanding than forming reputations through direct interactions, as it requires individuals to remember and recognise behaviours from third-party interactions. However, eavesdropping is crucial to providing animals with the capacity to predict the behaviour of others while avoiding the costs of direct experiences (Subiaul et al., 2008). Despite the importance of eavesdropping, only a few studies on non-human animals have investigated this behaviour and even fewer have indicated that a particular animal has the capacity for eavesdropping.

For example, Bshary and Grutter (2006) investigated eavesdropping in client–cleaner fish (*Labroides dimidiatus*) interactions. In this experimental study, client fish observed a cleaner and a client model (a fake fish) on opposite sides of a tank. On one side of the tank, mashed prawn was smeared on the client model resulting in the cleaner foraging on it. This made the cleaner appear to behave “cooperatively.” On the other side of the tank, another cleaner did not interact with a client model because prawn was not smeared on it; there was no client–cleaner interaction, so the client observer had no knowledge of the cleaner's cooperativeness. They found that the observing clients spent significantly more time next to the cooperative cleaner than the one with an unknown cooperation level, which suggests that the clients differentiated between the two cleaners and preferred the cooperative one. This was a clear demonstration of the fish's ability to eavesdrop. Although clients observed the cleaners interact with an inanimate client model, this study is impressive because it is the only controlled study to date that has investigated eavesdropping in animals performing natural behaviours, thus increasing the ecological validity of the results. Also, this was a conspecific-driven rather than experimenter-driven design. As it is difficult to control an animal's behaviour in an experiment, most studies on eavesdropping in animals involve interactions with humans. Thus, it would be logical to test species that are capable of acquiring information from humans, such as non-human apes (Bräuer et al., 2005) and dogs (*Canis lupus familiaris*) (Pongrácz et al., 2001).

Studies on eavesdropping typically involve animals observing human–human interactions in a begging situation, i.e., a generous person who gives food to a human beggar and a selfish person who refuses to give food. Russell et al. (2008) and Herrmann et al. (2013) tested chimpanzees, bonobos (*Pan paniscus*), and orangutans (*Pongo pygmaeus abelii*). Russell et al. (2008) tested gorillas (*Gorilla gorilla*) as well. These studies found that chimpanzees showed a significant preference for the generous person. A similar result was observed in orangutans in Herrmann et al. (2013) but not in Russell et al. (2008), and there was no significant preference by bonobos or gorillas. The positive results from the chimpanzees in these studies are contrasted by Subiaul et al. (2008, Experiment 1), where none of the seven chimpanzees showed a preference between the two humans. Subiaul et al. (2008, Experiment 3) conducted a follow-up experiment where chimpanzees observed humans interacting with a conspecific and found that three out of four chimpanzees chose the generous person on the first trial. However, these results should be considered with caution, as the sample size was small compared to Russell et al. (2008) and Herrmann et al. (2013), who tested 17 and 103 chimpanzees, respectively.

Enhancing the relevance of the interactions animals observe may be important in human–animal interaction studies on eavesdropping, as it could help them form a judgment of the humans. One potential confound to consider in such interspecific testing scenarios, however, is that these interactions are often highly artificial. In the wild, for instance, chimpanzee social interactions are exclusively between conspecifics, so chimpanzee–human eavesdropping studies, while informative, may lack ecological validity and should thus be interpreted with caution.

Eavesdropping on human–animal interactions might be more important for animals that live with humans, such as domesticated species, given that humans often provide them with valuable resources, such as food and shelter (Freidin et al., 2013). Thus, it would benefit them to form reputations of humans in order to choose the most appropriate person with whom to associate. Interestingly, results on domestic dogs and cats (*Felis silvestris catus*) have been mixed. In Rooney and Bradshaw (2006), dogs approached and preferred a person who won a tug-of-war game with a conspecific over the game's loser, suggesting that they were eavesdropping and formed a judgment that winners were desirable social partners. In contrast, Nitzschner et al. (2012) found that dogs showed no preference for a nice human, who interacted positively with a conspecific, or a human who ignored a conspecific. They only found a preference for the “nice” human compared to the “ignoring” human after the dogs had direct experience with them. Piotti et al. (2017) did not find evidence to support this, as dogs did not form a judgment of the experimenter based on her skilfulness or the quality of the interaction. Furthermore, a recent study on domestic cats indicated that they do not form reputations of humans based on direct experience, or on indirect interactions between humans and conspecifics (Leete et al., 2020). Thus, whether or not eavesdropping is a widespread phenomenon in non-human animals is still unknown (Table 1 summarises the studies that have been published to date).

**Table 1:** Summary of experiments on reputation formation in animals.

**Species**	**Interaction**	**Experience**	**Outcome**	**References**
Chimpanzees, bonobos, orangutans, gorillas	Human–human	Indirect	Chimpanzees: nice > mean Other apes: no significant difference	Russell et al., 2008
				Herrmann et al., 2013
Chimpanzees, bonobos, orangutans	Human–human	Direct Indirect	Nice > mean Chimpanzees and orangutans: nice > mean Bonobos: no significant difference	Experiment 1 Experiment 2
				Subiaul et al., 2008
Chimpanzees	Human–human Human–animal	Indirect Direct Indirect	No significant difference Learned to discriminate between generous and selfish Generous > selfish	Experiment 1 Experiment 2 Experiment 3
Dogs	Human–animal	Indirect	Winner > loser of tug-of-war game	Rooney and Bradshaw, 2006
				Nitzschner et al., 2012
Dogs	Human–animal	Direct Indirect	Nice > ignoring No significant difference	Experiment 1 Experiment 2
Dogs	Human–animal	Direct	No significant difference	Piotti et al., 2017
Cats	Human–animal	Indirect Direct	No significant difference	Leete et al., 2020

Apart from being the most studied species in eavesdropping research, chimpanzees and dogs are also among the most studied species in the field of comparative cognition in general. Chimpanzees are our closest living relatives and similar cognitive abilities in humans and apes would suggest common ancestry for complex cognition. Humans shaped the evolution of dogs through domestication (Hare and Tomasello, 2005), and looking at the social relationships between the species provides important clues about the effects of domestication on social abilities. However, in order to understand convergent evolution, it is important to study other evolutionarily distant animals that show similarities in cognition likely due to similarities in the environmental pressures they may have faced in their evolution. This field, known as convergent cognitive evolution, suggests behavioural flexibility, particularly in social problem-solving, may not be uniquely primate (Plotnik and Clayton, 2015). For example, elephants are an interesting taxon to test for eavesdropping because they are highly cooperative, large-brained, long-lived terrestrial mammals with a complex social organisation like chimpanzees (Byrne et al., 2009).

There is some evidence to suggest that elephants can form reputations of humans through direct experience. Bates et al. (2007) conducted a study in the Amboseli National Park, Kenya, where Maasai men demonstrate virility by spearing African elephants (*Loxodonta africana*), and Kamba agriculturalists pose little threat. They found that elephants showed greater fear when they detected the scent of garments previously worn by Maasai and reacted more aggressively to the red clothes that Maasai typically wear than to Kamba clothing. McComb et al. (2014) also found that elephants exhibited more defensive bunching and investigative smelling following playbacks of Maasai voices compared to Kamba voices. Furthermore, elephants exhibited these behavioural responses significantly more often when they heard the voices of Maasai men compared to Maasai women and Maasai boys. These results suggest that elephants had formed a bad reputation of Maasai men. Interestingly, Bates et al. (2007) found that elephants with no experience of spearing showed similar reactions as those that had interacted with Maasai men before. A possible explanation is that elephants had formed a bad reputation of Maasai men through indirect experience; however, this hypothesis would be very difficult to test experimentally with wild African elephants. Although there is little history of African elephants living in captivity in range countries, Asian elephants have a 4,000-year history of being tamed to live alongside humans in Asia. Thus, Asian elephants in Thailand—where more than 3,500 elephants live in captivity today—are ideal candidates for studying eavesdropping via human–animal interactions, as they are habituated to humans and interact with both familiar and unfamiliar people every day. They are also interesting to study from a comparative cognition perspective because they are not domesticated like dogs but have this long history of interacting with humans (Lair, 1997; Plotnik et al., 2013).

In the current study, we tested Asian elephants using an eavesdropping variation of the cooperative string-pulling task. Typically, in this test, two ends of a string must be pulled simultaneously to pull a platform baited with food rewards within reach. If only one end of the string, which is threaded around the platform, is pulled, the platform will not move and the string becomes unthreaded, making the baited platform inaccessible (Hirata and Fuwa, 2007). It is a well-established paradigm that has been used to test cooperation in many species, including Asian elephants (Plotnik et al., 2011), and has been used to study direct reputation formation in chimpanzees. After the chimpanzee subjects had experience using the apparatus with two conspecific partners that differed in their collaborative skills, they learned to recruit the more effective partner, which suggests that they had some understanding of the partner's role in the task (Melis et al., 2006). Although this task is often used to test cooperation between conspecifics, it has also been used to test cooperation between humans and dogs (Ostojić and Clayton, 2014) and wolves (Range et al., 2019a,b). Furthermore, in Experiment 2 of Range et al. (2019b), dogs and wolves were successful in recruiting a human partner to solve the cooperative string-pulling task.

Based on the elephants' experience with humans and their social complexity, as well as their success on the cooperative string-pulling task (Plotnik et al., 2011), we used, for the first time, the string-pulling task to investigate Asian elephants' ability to form reputations about cooperative and non-cooperative humans. The aim of the current study was to test whether Asian elephants can form reputations of humans through indirect and/or direct experience. In Experiment 1, we investigated whether elephants could differentiate between a cooperative and a non-cooperative partner in a string-pulling task. In Experiment 2, we simplified the design and investigated whether elephants could differentiate between a generous and a selfish partner in a begging situation, without the need for cooperation. We hypothesised that elephants derive and act on information about unfamiliar humans through reputation-like inferences after observing them interact with a conspecific and/or after directly interacting with them. Therefore, we predicted that elephants would significantly prefer the cooperative/generous partner over the non-cooperative/selfish partner. Direct reputation formation is a prerequisite for eavesdropping; thus, we predicted that elephants would at least show a significant preference for the cooperative/generous partner after direct experience.

## Experiment 1: String-Pulling Task

### Methods

#### Ethical Statement

This study was approved by the National Research Council of Thailand (Protocol #0002/848 and #0402/838). Ethical approval was obtained from the “Ethik und Tierschutzkommission” of the University of Veterinary Medicine Vienna (Protocol #ETK-15/12/2018) and Hunter College's Institutional Animal Care and Use Committee (Protocol #JP-Elephant Eavesdropping 1/22). The individual humans who participated in this study have either given written informed consent to publish photographs and videos containing their images in the Supplementary Materials, or we de-identified images by blurring faces when we were unable to get written informed consent.

#### Subjects

Twelve female captive Asian elephants (*Elephas maximus*) from the Golden Triangle Asian Elephant Foundation (GTAEF) living on the properties of the Anantara Golden Triangle Elephant Camp and Resort and the Four Seasons Tented Camp in Chiang Rai, Thailand, participated in the experiment between March and May 2019 (see Table 2). However, four elephants were excluded. One did not pass training (Lanna), one successfully obtained the food without the need for cooperation (Boonruam), and two were influenced by their mahouts (caretakers) in such a way that the elephants' choices may have been affected (Bo and Kumtoon). Each mahout is mostly responsible for a single elephant for extended periods of time, although during this study, mahouts did sometimes change due to circumstances beyond our control.

**Table 2:** List of subjects' participation.

**Elephant**	**Sex**	**Age (years)**	**Experiment 1**	**Experiment 2**
Bo^*^	F	41	Excluded (demonstrator)—mahout influence	Yes (demonstrator)
Boonruam	F	59	Excluded—obtained food without cooperation	No
Boonsri	F	51	Yes	Yes
Dah	F	17	Yes	Yes
Jathong	F	28	Yes	Yes
Kumtoon	F	15	Excluded—mahout influence	Yes
Lanna	F	31	Excluded—did not pass training	Yes
Mae Moo	F	55	No	Yes
Mae Noi	F	21	Yes	Yes
Bleum	F	21	Yes	Yes
Prae	F	29	Yes	No
Pumpui	F	42	Yes	No
Yui	F	27	Yes	No (demonstrator)
Riang Ngun	M	42	No (demonstrator)	Yes

* *Participated as an observer first and then acted as a demonstrator for half of the subjects after she had completed testing*.

#### Apparatus

The string-pulling apparatus was a 3 m (W) × 92 cm (L) tray with wheels attached to the bottom of the tray. The tray was placed on top of a 1.4 m (W) × 3 m (L) × 60 cm (H) metal frame with six adjustable legs. A single piece of a 16.5-m-long, 7-mm-thick rope was threaded through PVC pipe frames around the back and sides underneath the tray so that the loose ends appeared out of two openings on either side of the front of the apparatus, leaving 1.6 m of rope available in the testing area to pull upon approach. The apparatus was such that if only one end of the rope was pulled, the tray could not move, but rather the rope slid out of the frame and the non-pulled end of the rope became unavailable. Adjacent to each end of the rope, 2.2 m apart from each other, there were two clear buckets that extended 55 cm out of the tray. Hence, to successfully obtain the food, both ends of the rope needed to be pulled simultaneously to move the tray forward so that the buckets could pass under the volleyball net.

#### Experimental Setup

The experiment was conducted in a large field (10.6 m × 92 m) at the Anantara Golden Triangle Elephant Camp and Resort in Chiang Rai, Thailand. The two apparatuses were placed 2 m apart from each other and on one side of a 10-m volleyball net that was strung between two posts, forming a transparent but reliable barrier between the elephants and the apparatus (Plotnik et al., 2011). The elephants learned quickly that they were not allowed to pass beyond the barrier and thus stopped at the rope ends upon approach. The observer's area was partitioned by a red rope 5.2 m away from the volleyball net to create space so that the subject (hereafter also referred to as the observer) could not interfere with the interactions between the human partner and the demonstrator elephant during the observation phase.

**Figure 1:**
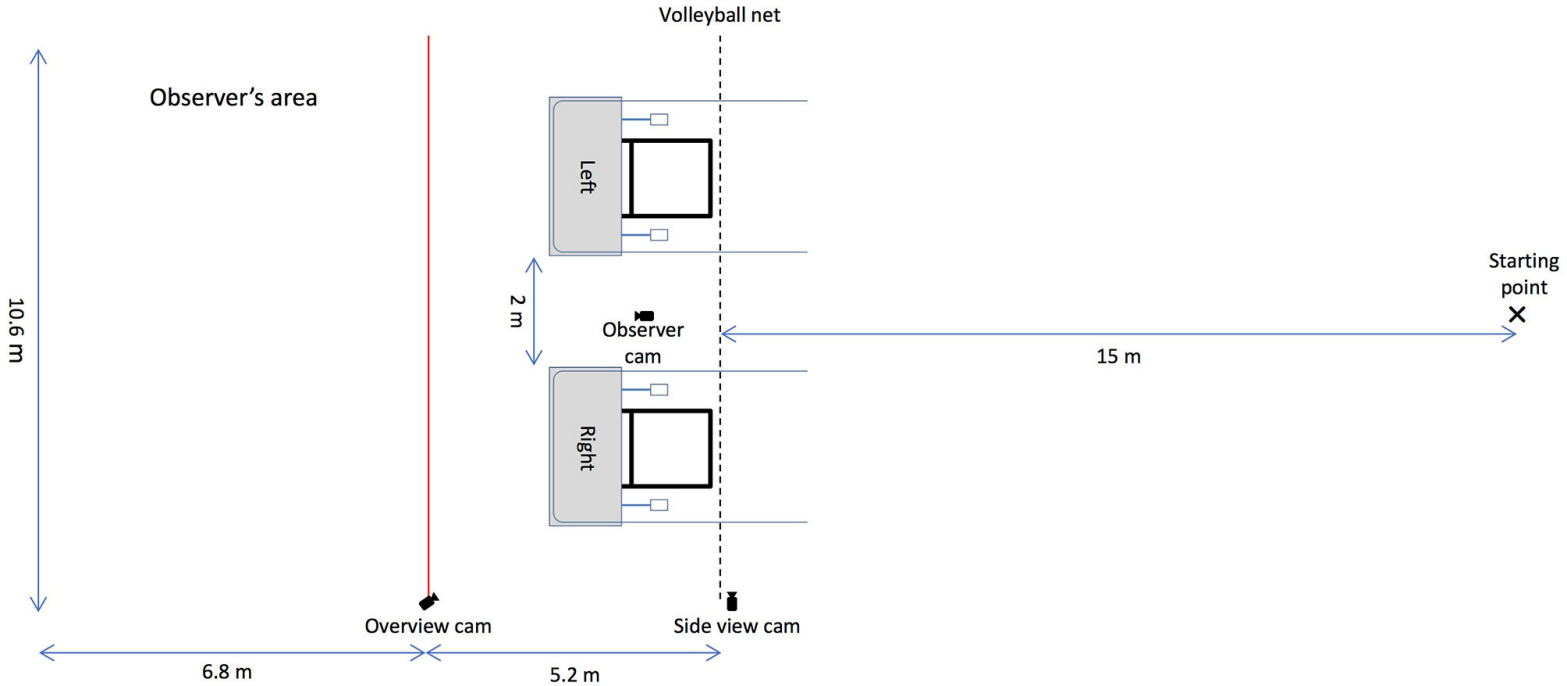
Schematic depiction of the setup of the string-pulling experiment. The two string-pulling apparatuses were placed 2 m apart from each other on one side of a volleyball net. In the baseline and the test phase, the subject stood at the starting point and each human partner stood in front and at the centre of one of the apparatuses. In the observation phase, the subject stood in the observer's area on the left (separated by a red rope) and the demonstrator stood at the starting point.

The whole experiment was recorded by three cameras. One Panasonic HD consumer video camera was placed on a tripod in between the two apparatuses facing the observer in a position that captured the whole observer's area. One GoPro Hero 4 Black was positioned by the volleyball net to gain a side view of the testing area, and another GoPro Hero 4 Black was placed on a tripod on a post that separated the observer's area and the apparatuses to gain a view of the whole testing area (see Figure 1).

#### Procedure

The experiment consisted of training sessions and two test sessions.

##### Training

It was important that each elephant was able to use the string-pulling apparatus reliably and understood that she could choose which apparatus to use in the test phase. Thus, the main experimenter trained all the subjects and the demonstrator elephant, which took between 2 and 11 sessions (*M* = 4.7, *SD* = 2.5). Each subject was required to successfully cooperate with a human who stood in front of one of the two baited apparatuses in 5 out of 6 trials before she could participate in the study.

##### Session 1

Throughout the experiment, two unfamiliar Thai human females acted as the partners—one wore white clothes and the other wore black. Asian elephants rely heavily on non-visual sensory information, such as auditory cues (Jacobson and Plotnik, in press), so each partner also said a predetermined sentence when they interacted with the demonstrator elephant to help the observer distinguish between the two partners visually and auditorily. Their role and colour of clothes were randomised and fixed within-subjects and counterbalanced between-subjects.

The male elephant (Riang Ngun) acted as the demonstrator for half of the subjects, and a female elephant (Bo) was selected to act as the demonstrator for the other half of the subjects. Each elephant was accompanied by a mahout for safety, who stood by the elephant and did not interact with him/her.

Prior to testing, the elephants could explore the environment freely for ~5 min to familiarise themselves with the location. The experiment consisted of three parts.

*Baseline* This phase was only conducted in Session 1 and tested whether the observer preferred one person prior to observing any third-party interactions with the demonstrator. Neither string-pulling apparatus was baited, and the ropes were placed on the tray behind the volleyball net so it was inaccessible to the elephant. Each human partner stood 5 m in front and at the centre of one of the apparatuses (the partners' positions were randomised). Each partner held a piece of food (apple or banana based on the elephant's preference) in their hands.

The elephant was positioned at a starting point marked by spray paint on the ground in the testing area (15 m away and equidistant from the partners). The mahout stood behind the elephant and released her to walk forward. When the elephant approached within arm's length of one of the partners, the first gave the food to the animal, followed immediately by the other calling the elephant to offer food. If the elephant did not approach either partner (e.g., she foraged or stood still), the mahout brought the elephant back to the starting point and released her again until a choice was made.

After this trial, the partners left the testing area, the observer was moved to the observer's area, and the demonstrator elephant was moved to the starting point in the testing area.

*Observation phase* First, the main experimenter only baited one of the string-pulling apparatuses (randomised and counterbalanced across subjects) and laid the two ends of the rope into the testing area. A partner stood in front and at the centre of it. The main experimenter then indicated to the mahout that the phase could begin. The mahout released the demonstrator from the starting point so he/she could walk forward and pull a rope. The demonstrator could choose which rope to pull, and then the partner moved to the opposite side of the apparatus. The observer then witnessed one of the following scenarios depending on the partner with whom the demonstrator interacted:

- Cooperative: when the demonstrator picked up the rope, the partner picked up the other end of the rope and said in Thai, “Let's eat!” in a friendly tone. They pulled the ropes simultaneously so that the tray moved forward and they both ate the food.
- Non-cooperative: when the demonstrator picked up the rope, the partner did not touch the apparatus and said in Thai, “I won't help!” in an unfriendly tone and walked away from the apparatus. Thus, the tray did not move forward and they were unsuccessful in obtaining the food.

**Figure 2:**
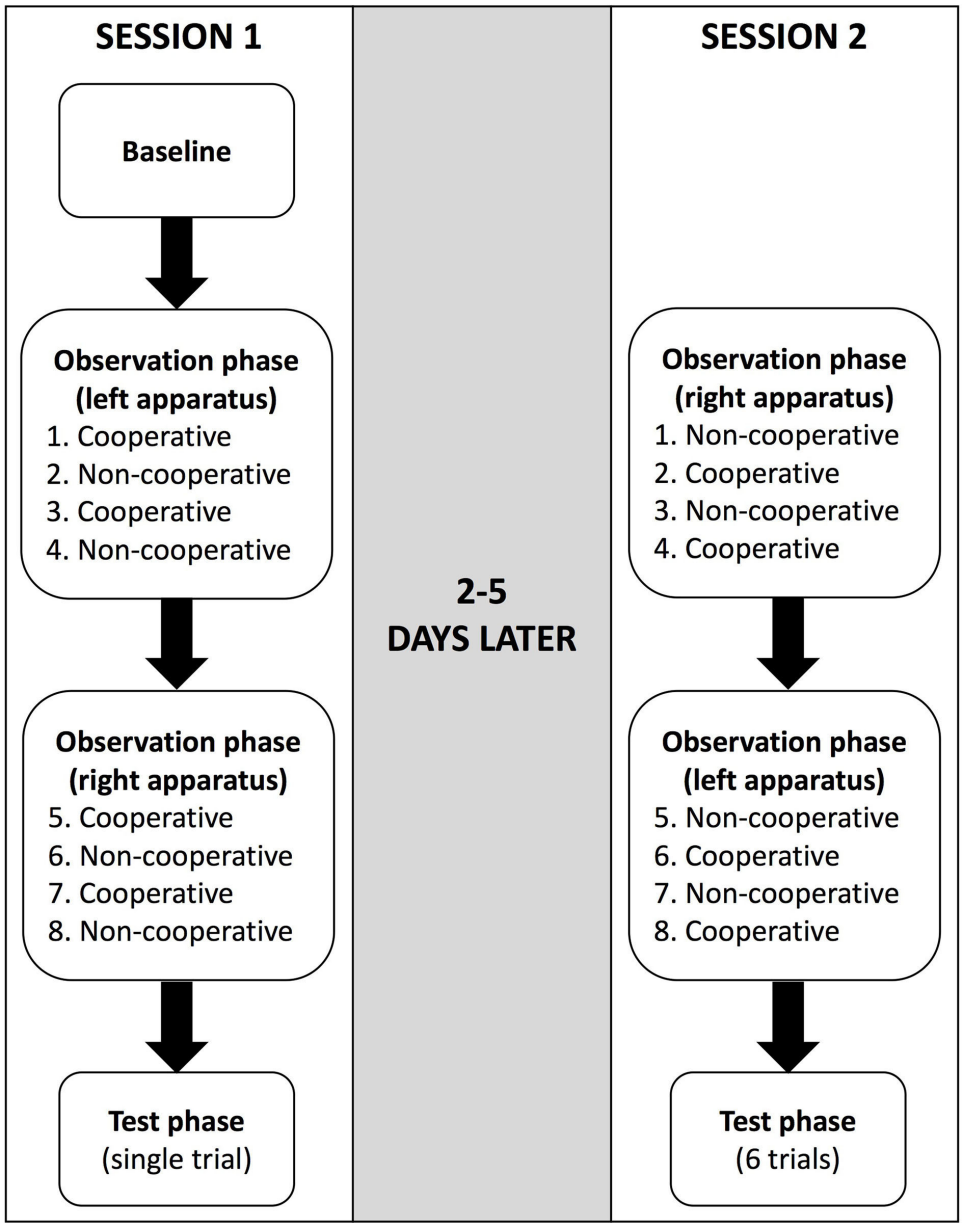
Flowchart illustrating an example of the procedure in the string-pulling experiment. The order of the string-pulling apparatus that was used first (left or right) and the partner who interacted with the demonstrator first (cooperative or non-cooperative) were randomised and counterbalanced between subjects.

After the interaction, the partner left the testing area, the main experimenter reset and re-baited the apparatus and the procedure was repeated with the second partner. After the demonstrator had interacted with each partner twice, the main experimenter placed the ropes on the tray behind the volleyball net, set up and baited the other apparatus, and then repeated the procedure. Overall, the demonstrator interacted with each partner twice alternately on one apparatus and twice alternately on the other apparatus (i.e., four interactions with each partner occurred in total—see Figure 2).

After the observation phase, the demonstrator left the testing area and the observer was moved to the testing area. The main experimenter set up and baited both apparatuses for the test.

*Test phase* Each partner stood directly in front and at the centre of one of the apparatuses (the partners' positions were randomised). As the observer stood at the starting point, the mahout stood behind and released the elephant to walk forward. When the elephant arrived at the apparatuses, she could choose a rope to pull and thus which partner with whom to cooperate. If the elephant did not approach a partner within 1 min, the mahout brought the elephant back to the starting point and the main experimenter added a handful of sunflower seeds into the buckets of each apparatus to increase her motivation. When the apparatuses were baited, the mahout released the elephant again until a choice was made or the mahout stopped the experiment if he felt the elephant did not want to participate anymore. This only occurred for one elephant (Pumpui) who did not complete the last trial (see Table 3).

**Table 3:** Results of the string-pulling experiment.

	**Eavesdropping subset**			**Reputation-learning subset**
**Elephant**	**Baseline**	**Session 1 (one trial)**	**Session 2**	**Session 2**
	**Did she choose the cooperative partner?**	**Did she choose the cooperative partner?**	**Did she choose the cooperative partner in the first trial?**	**No. of times she chose the cooperative partner**
Boonsri	Yes	Yes	Yes	2/6
Mae Noi	Yes	Yes	No	2/6
Yui	Yes	Yes	No	2/6
Dah	Yes	Yes	No	1/6
Pumpui	Yes	Yes	No	3/5
Prae	No	No	Yes	6/6
Bleum	Yes	No	Yes	3/6
Jathong	Yes	No	No	2/6

We defined a choice response by the rope the elephant touched. Once a choice had been made, the partner acted the same way as she did in the observation phase, i.e., the cooperative partner pulled the other rope and the non-cooperative partner moved away and did not touch the apparatus. After this trial, Session 1 was over and the partners and the observer left the testing area.

##### Session 2

The subject was tested 2–5 days later; she experienced the observation phase, where the order of the partners and the first apparatus used was counterbalanced, and six trials in the test phase. The partners' positions were semi-randomised such that they never stayed in the same position more than twice in a row.

#### Coding and Statistical Analyses

The observer must pay attention to the partner's actions to understand their different roles. Thus, we coded the subject's attention during the observation phase from the footage from the observer camera, which was synchronised with the footage from the overview and side view cameras and merged into one video.

We defined the beginning of the interaction as the moment the demonstrator touched the rope, and the end of the interaction as the time when the mahout asked the demonstrator to retreat and the partner left the testing area. During the cooperative interactions, we coded whether the subject was attentive at the moment when the demonstrator pulled the rope with the cooperative partner and/or while the demonstrator ate the food. During the non-cooperative interactions, we coded whether the subject was attentive at any time while the demonstrator pulled the rope.

We coded whether the subject was attentive or not in each interaction during the observation phase in both sessions. We defined the subject as being attentive towards the third-party interaction if the subject's head was oriented towards the interaction and her ears were out or her trunk was pointed towards the direction of the interaction. We defined the subject as not being attentive if her head and/or body were not oriented towards the interaction or she was attentive to the other partner. Furthermore, if the mahout interfered in any way that caused the subject to turn away from the interaction, point her trunk towards the mahout, or respond to a mahout direction, we coded her as not attentive.

All statistical analyses were carried out using R (version 3.6.2; R Core Team, 2019). HLJ and RD coded 20% of the videos for interobserver reliability, which was analysed using the intraclass correlation coefficient from the R package “irr” (version 0.84.1, Gamer et al., 2012) (ICC (two-way, agreement) = 0.717, *F* = 5.97, *p* < 0.001). HLJ and RD then each coded half of the videos.

We conducted generalised linear mixed-effects models (GLMMs) with binomial error structure and logit link function (McCullagh and Nelder, 1989), which were fitted using the function glmer of the R package “lme4” (version 1.1–21, Bates et al., 2015). Sixty-four observations were made with eight individuals.

We included attentiveness in the model by determining the proportion of interactions the elephants were attentive in the observation phase of both sessions. Then, we split the data into two subsets: the first subset, called “string-pulling eavesdropping” (comprising the baseline, the single trial in Session 1 and the first trial in Session 2), tested whether elephants formed a reputation of the humans based on their indirect experience; given the limited experience after Session 1, we argue that the first trial of Session 2 is still based on observation rather than the brief direct experience a few days prior. The second subset, called “string-pulling reputation-learning” (comprising the latter five trials), tested whether elephants formed a reputation of the humans based on their direct experience.

For the string-pulling eavesdropping subset, the full model included trial as a test predictor (factor with three levels), attentiveness (covariate) as a control predictor with fixed effects, and subject ID as a random intercept. The response variable was the subject's choice to approach the cooperative partner. Since we counterbalanced and hence controlled for the partners' roles, positions, and colour of clothes, we did not add those or demonstrator ID as control predictors in any of the analyses to reduce the complexity of the model.

For the string-pulling reputation-learning subset, the full model included z-transformed trial as a test predictor (covariate), attentiveness as a control predictor with fixed effects, subject ID as a random intercept and z-transformed trial as a random slope (Schielzeth and Forstmeier, 2009; Barr et al., 2013) within subject ID. To ease convergence, we changed the optimizer used by the function glmer to “bobyqa” (Jacobson, n.d.). We excluded the correlation between the random intercept and slope because it was estimated to be essentially 1, which is indicative of it not being identifiable (Matuschek et al., 2017).

We compared the full model to the null model which lacked trial (z-transformed in the string-pulling reputation-learning subset) in the fixed-effects part for both subsets. We determined the confidence of model estimates by means of a parametric bootstrap (function bootMeer of the package lme4). We assessed model stability by comparing the estimates obtained from the models based on all data with those obtained from models with the individuals excluded one at a time, which revealed the string-pulling eavesdropping subset to be very unstable (see the range of estimates in Supplementary Table 1) and the string-pulling reputation-learning subset to be unstable in some parts (see the range of estimates in Supplementary Table 2).

### Results

#### String-Pulling Eavesdropping

Five out of eight elephants chose the cooperative partner in the single trial of Session 1, and three out of eight elephants chose the cooperative partner in the first trial of Session 2. Only one elephant (Boonsri) chose the cooperative partner in the first two trials across the two sessions (i.e., the single trial of Session 1 and the first of six trials in Session 2—see Table 3).

The likelihood ratio test comparing the full and null model revealed that trial had a marginally non-significant effect (χ^2^ = 5.943, *df* = 2, *p* = 0.051), and attentiveness did not have a significant effect (χ^2^ = 2.884, *df* = 1, *p* = 0.089) on the elephants' choice to approach the cooperative partner. There was no significant difference in the elephants' choice for the cooperative partner between the single trial of baseline and the Session 1 single trial (*p* = 0.239, see Supplementary Table 1). Elephants were significantly less likely to choose the cooperative partner in the first trial of Session 2 compared to the baseline (*p* = 0.042, see Supplementary Table 1). We then re-levelled the factor trial such that the reference level was the single trial in Session 1 and found that there was no significant difference in the elephants' choice to approach the cooperative partner between the single trial of Session 1 and the first trial of Session 2 (*p* = 0.218, see Supplementary Table 1).

#### String-Pulling Reputation-Learning

The likelihood ratio test comparing the full and null model revealed non-significance for trial (χ^2^ = 0.892, *df* = 1, *p* = 0.345) and attentiveness (χ^2^ = 0.469, *df* = 1, *p* = 0.494) on the elephants' choice to approach the cooperative partner (see Supplementary Table 2). Thus, trial and attentiveness did not have a significant effect on the animals' choice to approach the cooperative partner in the latter five trials.

### Discussion

In Experiment 1, we compared the elephants' choice of the cooperative partner in the one baseline trial, the single trial of Session 1, and the first trial in Session 2 to analyse whether the elephants eavesdropped. They did not significantly choose the cooperative partner after observing the two humans interact with a conspecific (Session 1 single trial) compared to when they had no prior experience with them (baseline). However, we did find that elephants significantly chose the non-cooperative partner over the cooperative one in the first trial of Session 2 compared to when they had no prior experience with the partners (baseline). This result is surprising because the elephants chose the non-cooperative partner and so were unsuccessful in obtaining the food reward, which contrasts with our prediction. A closer look at our data reveals that the significant effect was due to 7 out of 8 elephants choosing the cooperative partner in the baseline and only 3 out of 8 choosing the cooperative partner in the first trial of Session 2. Therefore, the elephants did not choose the two partners equally at random in the baseline, which created a false-positive effect. Furthermore, five elephants chose the cooperative partner in the single Session 1 trial and three chose the cooperative partner in the first trial of Session 2, which are both close to chance level (four out of eight elephants). A high level of performance would be needed across all elephants (e.g., at least seven out of eight) for the result to be convincing for such a small sample size, thus we cannot make strong conclusions about eavesdropping in elephants from this result.

We compared the elephants' choice for the cooperative partner in the latter five trials in Session 2 to analyse whether the elephants demonstrated direct reputation formation. Overall, they did not significantly choose the cooperative partner more often than the non-cooperative partner and they did not learn to choose the cooperative partner after successive testing. Therefore, the results do not provide support for our hypothesis that elephants can form reputations of humans based on indirect or direct experience and attentiveness did not appear to be a factor. While the first trial of Session 2, like the only trial of Session 1, is based on observation, the additional five trials of Session 2 are based on direct experience. It is thus noteworthy that one elephant (Prae) chose the cooperative partner in every trial in Session 2. However, because she did not choose the cooperative partner in the single trial of Session 1 and no other elephants performed as consistently as she did in Session 2, it is not possible to determine whether she formed a reputation of the cooperative partner early in Session 2 or learned to choose that partner due to direct experience over the course of six successive trials.

**Figure 3:**
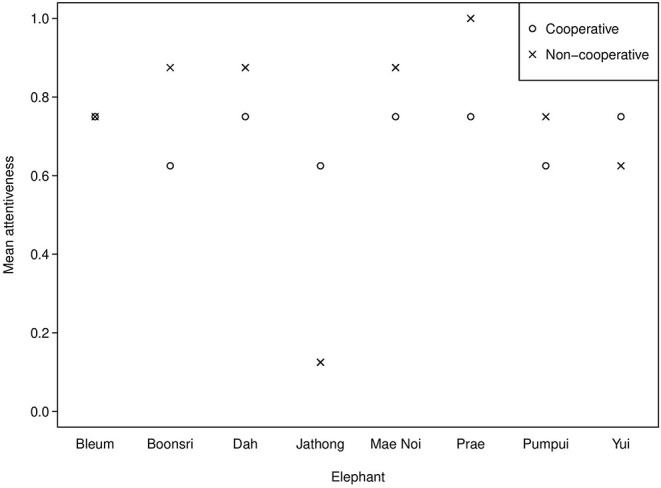
Mean attentiveness in the observation phase across Session 1 and 2 of the string-pulling experiment.

Russell et al. (2008) found that the top eight most attentive chimpanzees (54–80% attention, mean scores per subject over all trials) demonstrated eavesdropping, but the bottom eight (13–50% attention) did not. The behavioural coding reveals that the elephants in the present study demonstrated similarly high attentiveness (69–88%, mean scores per subject over all trials), except one elephant (Jathong), who scored 38% (see Figure 3). Prae was the most attentive, thus she may have demonstrated reputation formation. However, overall attentiveness did not have a significant effect on the elephants' choice for the cooperative partner, possibly due to our small sample size. An alternative explanation is that elephants may require different types of information to eavesdrop than chimpanzees. This could be because chimpanzees are more visual, while Asian elephants may be more reliant on acoustic and olfactory information in their environments (e.g., Plotnik et al., 2013, 2019; Ketchaisri et al., 2019); thus, they may need more non-visual sensory information to understand the partner's different roles.

Our findings are not in line with Melis et al. (2006), who found that chimpanzees recruited the effective partner through direct experience, and Range et al. (2019b), who found that dogs and wolves were successful in recruiting a human partner in the cooperative string-pulling task. A possible reason for the discrepancy between the results is that chimpanzees (Boesch and Boesch, 1989) and wolves (MacNulty et al., 2014) hunt cooperatively, whereas elephants forage individually. Moreover, it has been hypothesised that the cooperative skills of dogs with humans were inherited from their wolf ancestors (Canine Cooperation Hypothesis—Range and Virányi, 2015). As dogs were domesticated, they have a lot of experience with humans and heavily rely on them for food (Freidin et al., 2013), whereas captive Asian elephants are not domesticated and although they can cooperate with humans, they often work closely with a single individual, their mahout (Lair, 1997). Therefore, using the cooperative string-pulling task to investigate human–elephant interactions may lack ecological validity.

We used a variation of the cooperative string-pulling task to test eavesdropping because this paradigm has been used to test cooperation in elephants (Plotnik et al., 2011) and it seemed plausible that they would be flexible enough to generalise it to another context. In Plotnik et al. (2011)'s study, the elephants waited for their conspecific partner and did not pull the rope if their partners did not either. However, it is possible that, in the current study, the use of two different human partners may have made the task overly complex. The elephants may not have understood that one partner acted cooperatively and the other non-cooperatively.

Another potential explanation for these negative results is that the experimental apparatus itself may also have been too complicated. The two loose rope ends appeared out of openings on either side of the string-pulling apparatus, and the two apparatuses were placed side by side. Therefore, four rope ends were laid out at a similar distance to each other in the test phase. As the elephant was positioned in the centre between the two apparatuses at the start of each trial, it may have been difficult for the elephant to understand that the two central ropes were attached to different apparatuses when they approached, even though they had experience with the experimental setup in the test phase during training. We considered having the partners stand by the two central ropes so the elephant could only choose the outer ropes of each apparatus; however, this was not possible due to safety concerns.

For the reasons explained above, we conducted a follow-up experiment with a simpler design to test whether elephants can form reputation judgments of two humans—one generous and one selfish—after observing them interact with a conspecific and/or after directly interacting with them in a begging situation, like Subiaul et al. (2008, Experiment 3). This setup may also be more ecologically valid, as the elephants are often fed by unfamiliar people, such as tourists.

We also included two additional conditions in the follow-up experiment. As eavesdropping is defined as acquiring information through observing third-party interactions, we added an asocial control condition, where the partners acted “generously” and “selfishly” to an invisible third party. We predicted that, after observing third-party interactions, elephants would prefer the generous partner over the selfish partner in the experimental condition, but there would be no preference for either partner in the asocial control condition. Furthermore, the elephants did not have equal experience with the partners in Experiment 1, as their experience depended on who they approached in the test phase. Therefore, we added a “direct experience” condition to standardise their direct experience with each partner in the follow-up experiment. As direct reputation formation is a prerequisite for eavesdropping, we predicted that elephants would at least show a preference for the generous partner after direct experience.

## Experiment 2: Begging Situation

### Methods

#### Subjects

Ten captive Asian elephants, nine females and one male, from GTAEF participated in the experiment between August and September 2019 (see Table 2).

#### Materials and Experimental Setup

The experiment was conducted at the same location as Experiment 1; however, the string-pulling apparatuses were removed and a 5.7-m volleyball net was strung in the centre and perpendicular to the 10-m volleyball net. Three dots were marked on the ground on either side of the testing area; the bucket of food was placed on the central dot, which was marked 2.5 m away from the volleyball net. The human partners stood on each of two dots, one on either side of the centre dot/bucket and 1.1 m away from it. Additionally, some improvements were made to the testing area; two holding pens were built on opposite sides of the testing area to aid the elephants' starting point, two 10-m volleyball nets were strung one on top of the other on the left side of the field to prevent the elephants from foraging on bamboo adjacent to it, and a rope was tied to posts on the right side of the field to keep the elephants in the testing area.

**Figure 4:**
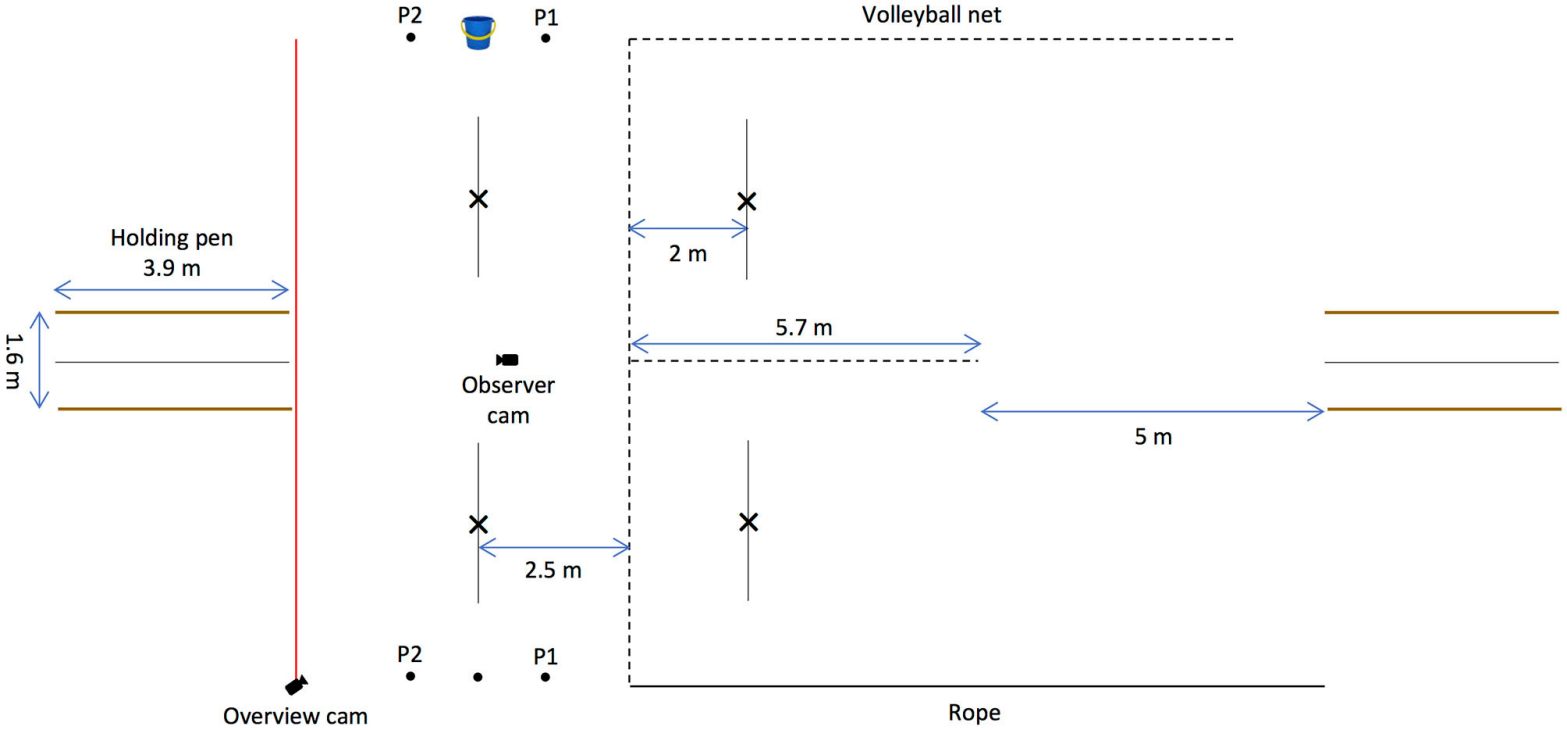
Schematic depiction of the setup of the begging experiment. A 5.7-m volleyball net was strung in the centre and perpendicular to the 10-m volleyball net. Three dots were marked on the ground on either side of the testing area; the bucket of food was placed on the central dot, and the human partners stood on either side of the bucket (P1 and P2). The crosses marked 2 m away on the right side of the volleyball net indicate where the human partners stood in the baseline and the test phase in the experimental and control conditions. The lines marked 2.5 m away on the left of the volleyball net indicate when the elephant made a choice response in the experimental and control conditions. In the direct experience condition, the experimental setup was mirrored. Two holding pens were built on opposite sides of the field to aid the elephants' starting point.

The whole experiment was recorded by one GoPro Hero 4 Black that was placed on a tripod 2 m behind the volleyball net facing the observer and another one that was placed on a tripod on a post to gain a view of the whole testing area (see Figure 4).

#### Experimental Design

**Figure 5:**
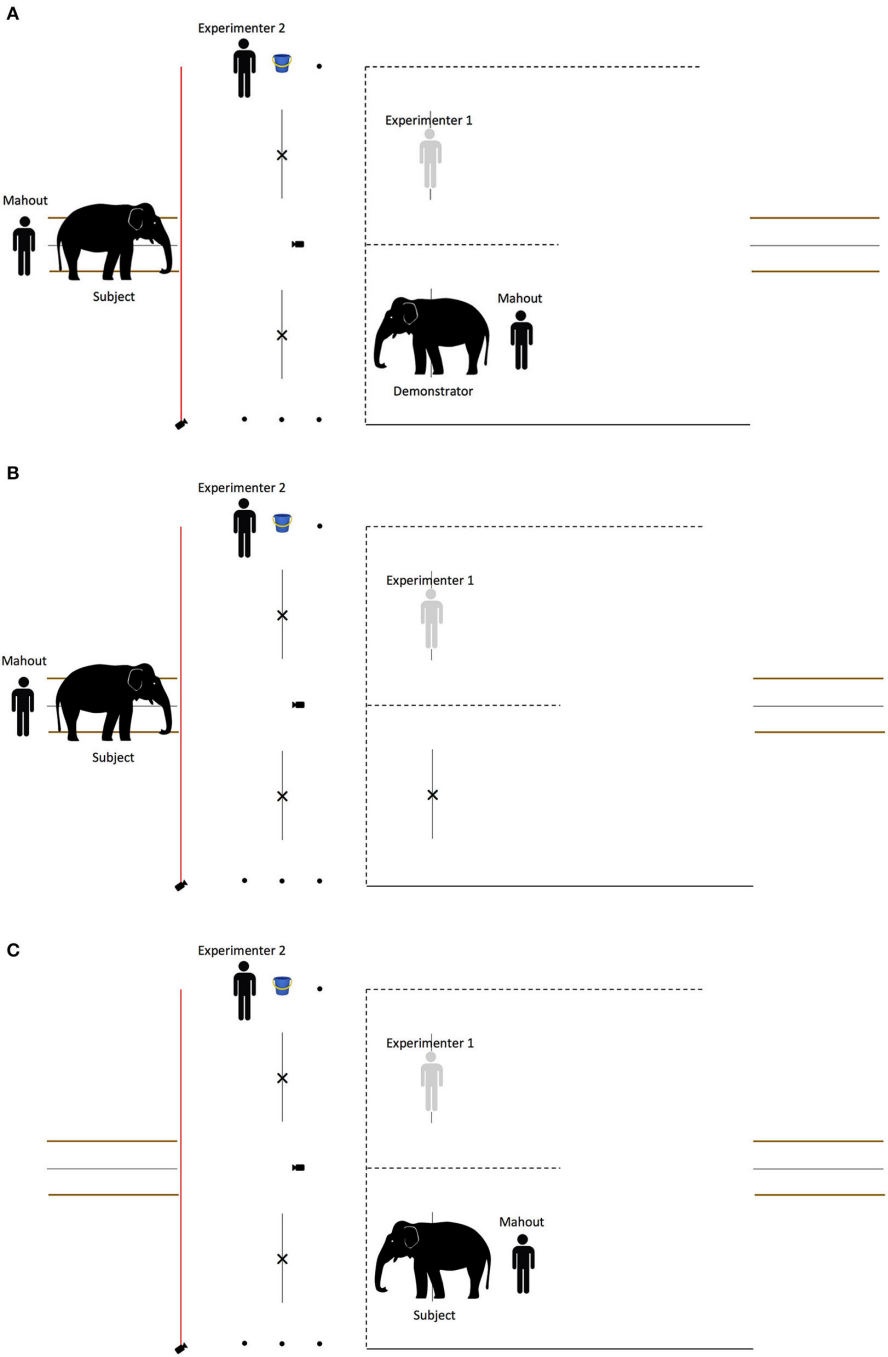
Schematic depiction of the observation phase in the begging experiment. In the experimental condition **(A)**, the subject observed the partners interact with the demonstrator. In the control condition **(B)**, the demonstrator was absent—the subject observed the partners perform the same actions without an elephant. In the direct experience condition **(C)**, there was no demonstrator or observer—the subject directly interacted with the partners.

There were three conditions (see Figure 5):

1. Experimental: the subject observed the partners interact with the demonstrator.
2. Control: the demonstrator was absent—the subject observed the partners perform the same actions without an elephant. The control was conducted so that if eavesdropping was observed in the experimental condition, we would be able to discern whether the elephants' responses were due to the partner observing the social interaction between partner and demonstrator, or whether the partners' actions *per se* (moving forward or away with a bucket of food) were sufficient to allow a discrimination between them.
3. Direct experience: there was no demonstrator or observer—the subject directly interacted with the partners. In this condition, the experimental setup was mirrored, i.e., the subject was in the holding pen in the testing area and the partners were on the other side of the volleyball net in the baseline and test phase.

This was a repeated-measures design; half of the sample experienced the experimental condition first, and the other half experienced the control condition first. All subjects experienced the direct experience condition after they were tested in the experimental and control conditions. There was a 4–12-day break between each condition.

#### Procedure

The procedure resembled that of Experiment 1 with some minor differences. The experiment consisted of two test sessions.

##### Session 1

Apart from the main experimenter and a research assistant, there were two human partners who were unfamiliar to the elephants in each condition. The pair of partners remained stable within conditions; there were a total of six human partners in the experiment, who were all Thai females. In the first condition, one partner wore white clothes and the other wore black. In the second condition, one partner wore a white spot-patterned poncho and the other wore a dark camouflage print poncho. In the direct experience condition that was conducted last, the partners wore white and black clothes again. As in Experiment 1, the partners said different, predetermined sentences when they interacted with the demonstrator to help the observer distinguish the two partners visually and auditorily. Their role and colour of clothes were randomised and fixed within-subjects and counterbalanced between conditions between-subjects.

A female elephant (Yui) acted as the demonstrator for half of the subjects, and another female (Bo) was selected to act as the demonstrator for the other half of the subjects after she had completed testing. In this experiment, mangoes were used to increase the elephants' motivation to participate, as it is a more high-value food reward than apples and bananas.

Prior to testing, the elephants could explore the environment freely for ~5 min to familiarise themselves with the location. The experiment consisted of three parts.

*Baseline* This phase was only conducted in Session 1. In the experimental and control conditions, the subject was placed in the holding pen in the observer's area. The partners stood in front of the volleyball net, 5 m away from each other; each person held a mango in their hands, and their positions were randomised.

The elephant's starting point was in the holding pen, 5.2 m away from the volleyball net; when the elephant was centred, the research assistant untied the red rope and the mahout stood behind the elephant and released him/her to walk forward. A choice response was made when the elephant approached a partner and was fed. The partner who was not chosen called the elephant forward to feed him/her. If the elephant did not approach either partner, the mahout brought the elephant back to the starting point and released him/her again until he/she made a choice.

After this trial, both partners left the testing area and the observer camera was set in place. The mahout moved the subject back to the holding pen, the rope was tied again, and the demonstrator was positioned on one side of the testing area (randomised and counterbalanced across subjects and conditions).

In the direct experience condition, the baseline was identical to the experimental and control conditions, but the experimental setup was mirrored. When the elephant approached a partner and was fed by her, the partner who was not chosen walked towards the elephant to feed him/her, as there was a volleyball net between them. After this trial, the subject was positioned on one side of the testing area (randomised and counterbalanced across subjects).

*Observation phase* In the experimental condition, the main experimenter placed a bucket of mangoes on one side of the testing area (randomised and counterbalanced across subjects) and the partners stood on either side of it (P1 and P2 in Figure 4), each holding a clear bucket. The demonstrator stood on the opposite side of the partition to the partners. The first partner (standing in P1) took a mango from the bucket, entered the testing area, and faced the demonstrator. The observer witnessed one of the following scenarios depending on which partner the demonstrator interacted with:

- Generous: the partner dropped the mango into the bucket and said in Thai, “Here you go!” in a friendly tone. Then she walked forward and put the bucket under the volleyball net so that the demonstrator could reach the food and eat it.
- Selfish: the partner dropped the mango into the bucket and said in Thai, “You can't have it!” in an unfriendly tone. Then she turned around and walked away from the elephant.

After the interaction, the partner walked to P2 and the second partner stood in P1. We controlled the partners' positions because P2 was closer to the subject in the observer's area. Hence, we ensured the time the partners spent close to the subject was equal to avoid the possible confound that elephants may simply choose the partner that spent more time close to them in the observation phase. The procedure was repeated with the second partner, and after the demonstrator had interacted with each partner twice, the partners and the demonstrator swapped sides and the main experimenter moved the bucket of food to the opposite side of the testing area. Overall, the demonstrator interacted with each partner twice alternately on each side of the volleyball net, thus there were four interactions with each partner in total. After the observation phase, the demonstrator left the testing area and the main experimenter removed the bucket of food and the observer camera. In the control condition, the observation phase was the same as the experimental condition; however, the demonstrator was not present, i.e., the partners “interacted” with an invisible demonstrator. In the direct experience condition, the observation phase was the same as the experimental condition; however, the demonstrator was not present and the observer was in the demonstrator's position, i.e., the partners interacted with the subject.

*Test phase* In the experimental and control conditions, the partners stood 2 m behind the volleyball net, each holding a clear bucket with a mango inside, and their positions were randomised. The mahout centred the elephant, then the research assistant untied the red rope and the mahout stood behind the elephant and released him/her to walk forward. If the elephant did not approach a partner within a minute, the mahout brought him/her back to the starting point and released him/her again until he/she made a choice or the mahout stopped the experiment if he felt the elephant did not want to participate anymore. This only occurred for one elephant (Mae Noi) who completed two trials in the experimental condition (see Table 4).

**Table 4:** Results of the begging experiment (experimental and control condition).

	**Condition**							
	**Experimental**				**Control**			
**Elephant**	**Baseline**	**Session 1**	**Session 2**	**Session 2**	**Baseline**	**Session 1**	**Session 2**	**Session 2**
	**Did he/she choose the generous partner?**	**(one trial)** **Did he/she choose the generous partner?**	**Did he/she choose the generous partner in the first trial?**	**No. of times he/she chose the generous partner**	**Did he/she choose the generous partner?**	**(one trial)** **Did he/she choose the generous partner?**	**Did he/she choose the generous partner in the first trial?**	**No. of times he/she chose the generous partner**
Dah	Yes	Yes	Yes	6/6	Yes	No	Yes	5/6
Bo	No	Yes	Yes	4/6	No	Yes	No	3/6
Bleum	Yes	Yes	Yes	2/6	Yes	Yes	Yes	6/6
Lanna	Yes	Yes	Yes	2/6	Yes	No	Yes	1/6
Kumtoon	No	No	Yes	5/6	Yes	No	No	1/6
Boonsri	No	No	No	3/6	Yes	Yes	Yes	3/6
Jathong	No	No	Yes	3/6	No	No	No	1/6
Mae Moo	No	No	No	3/6	No	Yes	No	1/6
Riang Ngun	Yes	No	No	2/6	No	No	No	3/6
Mae Noi	No	No	No	0/2	Yes	No	No	1/6

We defined a choice response as when the elephant crossed the line marked 2.5 m in front of the volleyball net. Once a choice had been made, the human partner acted the same way as she did in the observation phase, i.e., the generous partner walked forward to feed the elephant and the selfish partner turned around and walked away.

In the direct experience condition, the test phase was identical to the experimental and control conditions; however, the experimental setup was mirrored and a choice response was made when the elephant moved to one side of the volleyball net strung in the centre of the testing area. After this trial, Session 1 was over and the partners and the subject left the testing area.

##### Session 2

The subject was tested 2–5 days later; he/she experienced the observation phase, where the order of the partners was counterbalanced and the demonstrator and partners stood on the opposite side of the testing area from Session 1. There were six trials in the test phase, in which the partners' positions were semi-randomised so that they never stayed in the same position more than twice in a row.

#### Coding and Statistical Analyses

As in Experiment 1, we coded the subjects' attention from the footage from the observer camera, which was synchronised with the footage from the overview camera and merged into one video. We used the same ethogram to code whether the subject was attentive towards the interaction.

We defined the beginning of the interaction as when the partner stood facing the demonstrator before she dropped the food into the bucket, and the end of the interaction as when the partner moved to leave the testing area or after the demonstrator had eaten the food, whichever came last. During the interactions with the generous partner, we coded whether the subject was attentive during the moment of food exchange or when the demonstrator ate the food. During the interactions with the selfish partner, we coded whether the subject was attentive when she turned around to walk away from the demonstrator.

HLJ and RD coded 20% of the videos for interobserver reliability, which was analysed using the intraclass correlation coefficient from the R package “irr” (version 0.84.1) (ICC (two-way, agreement) = 0.873, *F* = 14.7, *p* < 0.001). HLJ and RD then each coded half of the videos.

Statistical analyses were also conducted in the same way as Experiment 1. We conducted GLMMs with binomial error structure and logit link function, which were fitted using the function glmer of the R package “lme4” (version 1.1–21). Two hundred and thirty-six observations were made with 10 individuals.

We split the data into two subsets. The first compared the experimental and control condition. In the experimental condition, the single trial in Session 1 and the first trial in Session 2 tested for eavesdropping. The justification for this interpretation is the same as in Experiment 1. In the control condition, the single Session 1 trial and the first trial in Session 2 tested whether the elephants' responses were due to the partner observing the social interaction between partner and demonstrator, or whether the partners' actions *per se* (moving forward or away with a bucket of food) were sufficient to allow a discrimination between them.

We included attentiveness in the model by determining the proportion of interactions the elephants were attentive in the observation phase of both sessions, and then we split the data into two further subsets: “begging eavesdropping” (comprising the baseline, the single trial in Session 1 and the first trial of Session 2 in the experimental and control condition) to test whether elephants formed a reputation of the humans based on their indirect experience, and “begging reputation-learning” (comprising the latter five trials in the experimental and control condition) to test whether elephants formed a reputation of the humans based on their direct experience.

The second subset analysed the direct experience condition separately, which we refer to as the “direct experience” subset. In this subset, we also split the data into two further subsets: Session 1 (comprising the baseline and single trial) tested whether elephants formed a reputation of the humans based on four brief interactions and Session 2 (comprising all six trials) tested whether elephants need more interactions, across two separate days, to form a reputation of the humans.

For the begging eavesdropping subset, the test predictors were trial (factor with three levels) and condition (factor with two levels). The control predictors were condition order (factor with two levels) and attentiveness (covariate). Therefore, the full model included an interaction between trial × condition × condition order, attentiveness as a fixed effect, and subject ID as a random intercept.

The full model for the begging reputation-learning subset included an interaction between z-transformed trial × condition × condition order and all lower order terms this encompasses, attentiveness as a fixed effect, subject ID as a random intercept, and z-transformed trial as a random slope within subject ID.

To ease convergence, we fitted both models using the optimizer “bobyqa.” Then, we compared the full model to the null model for both sessions, from which we removed trial (z-transformed in the begging reputation-learning subset), condition, and the interactions in which they were involved.

For Session 1 in the direct experience subset, the full model included trial as a test predictor (factor with two levels) and subject ID as a random intercept. To ease convergence, we used the optimizer “bobyqa.” Then, we compared the full model to the null model, where trial was removed from the fixed effects part of the model.

For Session 2 in the direct experience subset, the full model included z-transformed trial as a test predictor (covariate), subject ID as a random intercept, and a z-transformed trial as a random slope within subject ID. To ease convergence, we used the optimizer “bobyqa.” Then, we compared the full model to the null model, where z-transformed trial was removed from the fixed effects part of the model.

We assessed model stability by comparing the estimates obtained from the models based on all data with those obtained from models with the individuals excluded one at a time. This revealed that the model for the begging eavesdropping subset was very unstable (see the range of estimates in Supplementary Table 3). The model for the begging reputation-learning subset was also, in some parts, very unstable (see the range of estimates in Supplementary Table 4). The model for Session 1 of the direct experience subset had good stability. However, the model had extremely large estimates (see Supplementary Table 5) and a large standard deviation estimated for the random effect of individual (*SD* = 36.517), likely due to lack of data; thus, the model is not trustworthy. For Session 2 of the direct experience subset, the model was moderately stable (see the range of estimates in Supplementary Table 6).

### Results

As previously outlined, each condition (experimental and control) consisted of two sessions. The first two trials across the two sessions (i.e., the single trial of Session 1 and the first of six trials in Session 2) of the experimental condition compared to those of the control condition test for eavesdropping. The latter five trials in Session 2 test for direct reputation formation. The presentation order of experimental and control conditions was counterbalanced.

#### Begging Eavesdropping

In the single trial of both the experimental and control conditions in Session 1, four out of 10 elephants chose the generous partner. In Session 2, six elephants chose the generous partner in the first trial of the experimental condition and four elephants chose the generous partner in the first trial of the control condition. Four elephants chose the generous partner in the first two trials in the experimental condition, and two elephants chose the generous partner in the first two trials in the control condition. Only one elephant (Bleum) chose the generous partner in both trials and in both conditions. Additionally, Bleum chose the generous partner in every trial in both sessions of the control condition and another elephant (Dah) chose the generous partner in every trial in both sessions of the experimental condition (see Table 4).

The likelihood ratio test comparing the full and null model did not reveal significance for attentiveness (χ^2^ = 0.056, *df* = 1, *p* = 0.812) or the interaction between trial × condition × condition order (χ^2^ = 0.726, *df* = 2, *p* = 0.696). Thus, none of the predictors had a significant effect on the animals' choice to approach the generous partner (see Supplementary Table 3).

#### Begging Reputation-Learning

The likelihood ratio test comparing the full and null model also did not reveal significance for attentiveness (χ^2^ = 0.194, *df* = 1, *p* = 0.660) or the interaction between trial number × condition × condition order (χ^2^ = 1.992, *df* = 1, *p* = 0.158). Thus, none of the predictors had a significant effect on the animals' choice to approach the generous partner (see Supplementary Table 4).

#### Direct Experience

Six out of 10 elephants chose the generous partner in the Session 1 single trial, and only one elephant (Lanna) consistently chose the generous partner in all six trials in Session 2 (see Table 5).

**Table 5:** Results of the begging experiment (direct experience condition only).

**Elephant**	**Baseline**	**Session 1 (one trial)**	**Session 2**
	**Did they choose the generous partner?**	**Did they choose the generous partner?**	**No. of times they chose the generous partner**
Dah	Yes	Yes	2/6
Bo	No	Yes	2/6
Bleum	No	Yes	4/6
Lanna	No	No	6/6
Kumtoon	No	Yes	2/6
Boonsri	No	Yes	3/6
Jathong	No	No	2/6
Mae Moo	Yes	Yes	1/6
Riang Ngun	No	No	4/6
Mae Noi	No	No	4/6

For Session 1, the likelihood ratio test comparing the full and null model revealed significance (χ^2^ = 8.881, *df* = 1, *p* = 0.003), indicating that elephants were significantly more likely to choose the generous partner in the Session 1 single trial compared to the baseline (*p* = 0.008, see Supplementary Table 5). However, as stated in the methods, the model was very unstable, likely due to lack of data, and thus the model is not trustworthy. Therefore, we are unable to interpret this result.

For Session 2, the likelihood ratio test comparing the full and null model did not reveal significance (χ^2^ = 0.098, *df* = 4, *p* = 0.755). Therefore, trial had no significant effect on the animals' choice to approach the generous partner.

As the results were non-significant for both experiments, we tested whether the elephants had a side bias. We conducted a Heterogeneity G-test (McDonald, 2014) for 10 out of 14 elephants that completed more than one condition (the four elephants who only participated in the string-pulling experiment were excluded from the analysis). We found that there was no side bias across conditions for either the left or the right side (*Gh* = 3.969, *df* = 9, *p* = 0.913, *Gt* = 4.00, *df* = 10, *p* = 0.947).

### Discussion

In Experiment 2, we found that the elephants did not differentiate between the generous or selfish partner in a begging situation after indirect or direct experience. Although we found that elephants significantly preferred the generous partner after four brief interactions with the two partners (the single trial in Session 1) compared to when they had no prior experience with them (baseline), a closer look at our data reveals that the significant effect was due to eight out of 10 elephants choosing the selfish partner in the baseline and six out of 10 choosing the generous partner in the single trial in Session 1. Therefore, as in Experiment 1, the elephants did not choose the two partners equally at random in the baseline, which again created a false-positive effect. The number of elephants that chose the generous partner in Session 1 (six out of 10 elephants) was also close to chance level (i.e., five out of 10 elephants). A high level of performance would be needed across all elephants (at least eight out of 10) for the result to be convincing for such a small sample size; thus, we cannot make strong conclusions about eavesdropping in elephants from this result. Furthermore, the elephants did not differentiate between the generous or selfish partner after direct experience across two separate days. Therefore, the results do not support the hypothesis that elephants can form reputations of humans.

**Figure 6:**
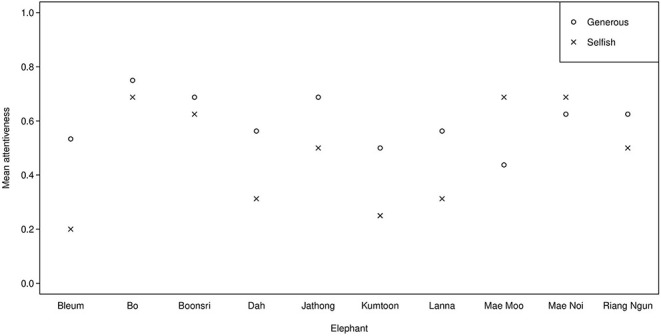
Mean attentiveness in the observation phase across Session 1 and 2 for the experimental and control condition of the begging experiment.

We ruled out lack of attentiveness as a possible explanation for why we did not find evidence of eavesdropping, as attentiveness had no significant effect on the animals' choice to approach the generous partner. However, the elephants appeared to be somewhat less attentive in this experiment (37–72% attention, mean scores per subject over all trials) (see Figure 6) compared to Experiment 1 (38–88%) (see Table 6). Out of the five elephants that participated in both experiments, four elephants' attentiveness scores decreased. A possible explanation is the fatigue effect; as half of the subjects had already been tested in a similar setup in Experiment 1, it is possible that they learned that food was rewarded at 50/50 chance in the test phase. If they learned this, and such rewards were sufficient motivation to participate, there may have been little additional motivation to pay attention to the observation phase in this experiment.

**Table 6:** Mean attentiveness scores per subject over all trials in the observation phase in Session 1 and Session 2 in both experiments (DP = did not participate at all in this experiment; E = data were excluded from analysis due to mahout interference).

	**Experiment 1: String-pulling task**		**Experiment 2: Begging situation**	
**Elephant**	**Attentiveness**	**Interactions scored (out of 16)**	**Attentiveness**	**Interactions scored (out of 32)**
Prae	88%	16	DP	NA
Dah	81%	16	44%	32
Mae Noi	81%	16	66%	32
Boonsri	75%	16	66%	32
Bleum	75%	16	37%	30
Jathong	38%	16	59%	32
Pumpui	69%	16	DP	NA
Yui	69%	16	DP	NA
Bo	E	NA	72%	32
Mae Moo	DP	NA	56%	32
Riang Ngun	DP	NA	56%	32
Lanna	DP	NA	44%	32
Kumtoon	E	NA	37%	32

The Experiment 2 attentiveness scores of the three elephants that did not participate in Experiment 1 were lower than the Experiment 1 attentiveness scores of four of the five elephants whose data were included from both experiments (see Table 6). Thus, these results cannot be explained by the fatigue effect. This may be because the string-pulling apparatus used in Experiment 1 made a loud noise when the tray moved forward, which may have caught the elephants' attention. In this experiment, the main auditory cue to distinguish between the partners was their voices when they spoke to the demonstrator, but the action of walking towards or away from the demonstrator was the crucial information the subjects needed to attend to in order to distinguish the partners' roles. The partners did not speak before the test trials—if they had, it may have helped the elephants to differentiate between the partners and approach the generous partner based on the location of the speaker. Therefore, the elephants may not have been able to distinguish the partners' roles in this experiment based on the primarily visual information and the limited auditory information.

## General Discussion

The results indicate that Asian elephants did not differentiate between a cooperative and a non-cooperative partner in a cooperative string-pulling task, nor did they differentiate between a generous and a selfish partner in a begging situation. We ruled out lack of attentiveness and side bias as possible explanations for the results in both experiments; thus, our results do not support the hypothesis that elephants can form reputations of humans. However, given the small sample size in our study and the poor model stability for the analysis of the experiments, we reach this conclusion with caution. Furthermore, as we discuss below, based on our knowledge of elephants in captivity, particularly in Southeast Asia, as well as previous research on African elephants in the wild, we believe further research that takes the elephants' multimodal sensory perception into account (Jacobson and Plotnik, in press) may yield different results.

Our results are not in line with Subiaul et al. (2008), which found some evidence for reputation formation through direct (Experiment 2) and indirect (Experiment 3) experience in chimpanzees. In Experiment 2 of Subiaul et al. (2008), five out of seven chimpanzees learnt to discriminate between the generous and selfish partners after 15–75 direct experiences, and one of those failed to maintain a preference for the generous partner. In our study, the elephants only had eight direct experiences with each partner over two sessions; thus, we may have found evidence for direct reputation formation if the elephants had had more direct experience. In Experiment 3 of Subiaul et al. (2008), three chimpanzees chose the generous partner on the first trial and one developed a preference after successive testing. Taken together, it is difficult to conclude that the chimpanzees demonstrated reputation formation based on these results, as the sample size was very small.

Direct reputation formation is a prerequisite for eavesdropping, and as the elephants did not demonstrate reputation formation even after direct experience, it is unsurprising that we did not find evidence of eavesdropping. However, it is unusual that we did not find evidence of direct reputation formation. A possible explanation is that elephants cannot form reputations of humans, but this is highly unlikely. Previous research has shown that African savanna elephants are able to distinguish between humans after direct experience based on visual, olfactory, and auditory cues (Bates et al., 2007; McComb et al., 2014). The relationship between individual humans and Asian elephants in captivity throughout Southeast Asia also makes it highly unlikely that they cannot form reputations of people. Elephants in logging or tourist camps often work closely with a specific human mahout over many years and not only recognise and respond to that individual mahout but also have been known to respond differentially to other mahouts, veterinarians, or managers (Lair, 1997).

There are also several differences between the earlier studies (Bates et al., 2007; McComb et al., 2014) and the present study that may explain the discrepancy in the results. First, it is important to acknowledge that Asian and African savanna elephants are different species with different ecologies. Recent research comparing elephants' ability to follow human-provided social cues has found differences between African (Smet and Byrne, 2013) and Asian elephants (Plotnik et al., 2013; Ketchaisri et al., 2019); therefore, it is possible that there are significant species-level differences between African and Asian elephant behaviour and ecology (Ketchaisri et al., 2019). Second, the previous research focused on wild African elephants, where interactions with Maasai men may be costly; thus, it was crucial for the elephants' survival to eavesdrop in that context. If the elephants chose the non-cooperative or selfish person in the present study, there was a small cost of not receiving a small food reward. Therefore, it is likely that elephants can form reputations of humans, but our experimental designs lacked ecological validity and thus the elephants did not respond as they would in a context with which they were familiar (either in the wild or captivity). It would be interesting to test reputation formation using human–elephant interactions in a more ecologically valid non-foraging context, for example, farmers who react differently to wild Asian elephants raiding their croplands, or a similar experiment to the present one with humans interacting with captive Asian elephants in a helping situation.

Another explanation for the negative results is that the elephants in the present study may not have been motivated to choose the cooperative or generous partner for a small food reward. Humans provide much of the food for captive Asian elephants, but the elephants also spend some time during the day foraging for themselves. Domesticated species such as dogs rely heavily on humans for food and food rewards (Freidin et al., 2013); thus, a food-sharing situation may be more relevant for dogs. Nevertheless, we tried to test the elephants at their maximum motivation by conducting the experiments early in the morning before they were fed by their mahouts or tourists and while the temperature was not too hot. We also tried to increase their motivation by feeding them high-value foods, such as mangoes, bananas, apples, and sunflower seeds. We could not restrict their diet the night before to increase their motivation, which can be done with other species such as dogs. Elephants eat roughly 250 kg of food a day; thus, restricting their diet for any length of time is neither practical nor ethical.

A limitation of the present study is that we could not be certain whether the elephants were paying attention to the third-party interactions in the observation phase. Although we operationalised attentiveness, it is difficult to define attentiveness in elephants. Therefore, although the interobserver reliability for our behavioural coding was based on specific behavioural measures, we could not be certain that the elephants were paying attention to the observations. While the elephants may have been paying attention during trials, they may have been watching the conspecific or the food rather than the human partner's actions/identity, which is necessary for them to understand the partners' different roles. Previous research on dogs showed that they did not develop a preference for the generous person even after many direct experiences because they were too focused on the food (Nitzschner et al., 2012). Thus, if the elephants were not watching the partners' actions, this would explain why they did not differentiate between the partners.

Another limitation is that our experiments were mainly visual tasks and elephants rely on more non-visual sensory information, such as auditory and olfactory cues (Plotnik et al., 2013, 2014, 2019; Schmitt et al., 2018, 2020; McArthur et al., 2019; Jacobson and Plotnik, in press). The elephants did have access to complementary visual, acoustic, and olfactory cues in our study; apart from the individuals' smell, the partners wore contrasting clothes and said different sentences when they interacted with the demonstrator. Furthermore, there was an audible noise when the apparatus moved in the string-pulling experiment and whenever food was placed into the buckets in both experiments. However, these cues may not have been salient enough for the elephants to distinguish between the two partners. The majority of studies on eavesdropping in animals to date have used visual tasks, as it is easiest to distinguish the partners' roles by having them perform different actions, and it makes sense to use this kind of experimental setup to test visual animals such as dogs, cats, and primates. However, we may learn more about eavesdropping in elephants if future research is designed using primarily auditory information, such as a playback experiment.

We used GLMMs to analyse whether the elephants' choice for the cooperative/generous partner differed before and after the observation phase, and our results suggest some issues with this analysis and our study design. We conducted a choice test because this seemed to be the best way to measure eavesdropping in elephants. Some previous studies have used proximity to an individual to measure eavesdropping in animals (e.g., Bshary and Grutter, 2006; Russell et al., 2008; Nitzschner et al., 2012; Leete et al., 2020). This is a particularly good measure for dogs as they tend to seek human contact, but it is not a good measure for elephants because they would be unlikely to approach a human unless food was involved. Consequently, a choice test can only be used once or twice to measure eavesdropping, as any more experience with the partners may lead to direct reputation formation. We were only able to test 8–10 elephants, so there were only 16 data points in the first experiment (string-pulling task) and 40 data points in the second experiment (begging situation with two conditions). We found that elephants did change their choice after the observation phase, but these were found to be false positives because the elephants did not choose equally at random in the baseline, and ultimately, the critical analyses came down to examination of the raw data. Thus, conducting a GLMM may be problematic if there are not enough data to measure the response accurately. In situations where a larger sample size is not possible, it might be worth considering alternative means of evaluating and presenting data, such as a single-case multiple-baseline AB design. With this design, the outcome variable is measured on a few “cases” (e.g., subjects) in a baseline phase (the A phase) and in an experimental phase (the B phase) (Bulté and Onghena, 2009; Bouwmeester and Jongerling, 2020; Levin et al., 2020). Then, the effect of the experimental phase is analysed using visual inspection of the pattern of observations in each phase and randomisation tests. As each case serves as its own control, this design may allow researchers to be more confident that the observed effect is attributed to the change in phase rather than extraneous variables and thus might be a good choice for future studies.

We had to remove several control variables from the GLMMs to reduce complexity, e.g., experimenter ID, role of experimenter, the colour of the experimenter's clothes, and experimenter position. Although we randomised and counterbalanced these variables, it would have been better to include them in the model or change the study design so that these variables did not need to be controlled for in the model, e.g., if each subject was tested with different pairs of individuals as experimenters. Finally, all previous studies on eavesdropping did not include a baseline to test whether there was a preference for an experimenter (e.g., Russell et al., 2008; Subiaul et al., 2008; Nitzschner et al., 2012; Herrmann et al., 2013; Piotti et al., 2017). Therefore, they assumed that the animals did not prefer an experimenter before the observation phase and their initial choice would be at chance level, i.e., 50%. However, our study shows that this is an important consideration—had we not conducted the baseline and included it in the analysis, we may have interpreted the results very differently. Therefore, we suggest that future experiments should have a larger sample size if possible, control for confounding variables, and include a baseline.

In conclusion, we tested whether Asian elephants can form reputations of humans in two different paradigms: (1) a cooperative string-pulling task and (2) a begging situation. Although we did not find evidence to support our hypothesis that elephants can form reputations of humans after indirect or direct experience, our study aids in our understanding of human–elephant interactions and informs our development of future species-specific research paradigms that focus on ecological validity within the socio-cognitive domain. Our research highlights the importance of considering sensory perception in socio-cognitive tasks, particularly those involving interspecific interactions. Further research on eavesdropping and reputation formation is needed because it could help explain how knowledge about humans spreads socially in elephants. In addition, a greater understanding of the role of elephant cognition in the elephant's interactions with humans could have important implications for improving captive elephant management, particularly as it relates to the management of mahout–elephant relationships. Finally, the flexibility of the elephant's decision-making process, particularly as it pertains to their decisions regarding whether to interact with specific humans, could be relevant for mitigating the increasing conflict between wild elephants and humans due to habitat loss in elephant range countries.

## Data Availability Statement

The original contributions presented in the study are included in the article/Supplementary Materials, further inquiries can be directed to the corresponding author/s.

## Ethics Statement

This study was reviewed and approved by the National Research Council of Thailand (Protocol #0002/848 and #0402/838). Ethical approval was obtained from the Ethik und Tierschutzkommission of the University of Veterinary Medicine Vienna (Protocol #ETK-15/12/2018) and Hunter College's Institutional Animal Care and Use Committee (Protocol #JP-Elephant Eavesdropping 1/22).

## Author Contributions

HLJ, FR, SMP, RD, and JMP designed the study and refined the methodology. HLJ conducted the experiments. HLJ and RD coded the videos. HLJ analysed the data and drafted the manuscript. FR, SMP, RD, and JMP contributed to the analyses and to the writing of the manuscript. All authors contributed to the article and approved the submitted version.

## Conflict of Interest

The authors declare that the research was conducted in the absence of any commercial or financial relationships that could be construed as a potential conflict of interest.
