# Supplementary material for: Investigating Indirect and Direct Reputation Formation in Asian Elephants (Elephas maximus)
Source: Front Psychol. 2021 Jan 8;11:604372. doi: 10.3389/fpsyg.2020.604372 (PMC7841644; doi:10.3389/fpsyg.2020.604372)
Supplement: Supplementary file 1 [file Table_1.docx]

Supplementary Material

# Supplementary Data

**S1 Dataset. Experiment 1: String-pulling task.** Trial 0 refers to the baseline, trial 1 refers to the single trial in Session 1 and trials 2-7 refer to the six trials in Session 2. Choice represents the subject’s choice with whom to cooperate; ‘coop’ refers to the cooperative partner, ‘noncoop’ refers to the non-cooperative partner. Attentiveness was determined by the proportion of demonstrations the subject was attentive in the observation phase of each session.

**S2 Dataset. Experiment 2: Begging situation.** Order refers to the order of conditions the subject experienced; ‘ec’ refers to experimental condition first, ‘ce’ refers to control condition first. Trial 0 refers to the baseline, trial 1 refers to the single trial in Session 1 and trials 2-7 refer to the six trials in Session 2. Attentiveness was determined by the proportion of demonstrations the subject was attentive in the observation phase of each session.

**S3 Dataset. Subjects’ side preference data.** We analyzed the subjects’ choice to approach the partner standing on the left or the right in the test phase for 10 out of 14 elephants that completed more than one condition over both experiments.

**S1 Video. Example of the procedure for Experiment 1.** The video shows the baseline, an interaction with the cooperative partner (wearing white), the main experimenter resetting and re-baiting the apparatus, then an interaction with the non-cooperative partner (wearing black) in the observation phase, and the test phase in Session 1. In the observation phase, the top left window shows footage from the overview camera and the top right window shows footage from the observer camera. In the test phase, the top right window shows footage from the side view camera.

**S2 Video. Example of the procedure for the experimental condition in Experiment 2.** The video shows the baseline, an interaction with the selfish partner (wearing white) and the generous partner (wearing black) in the observation phase, and the test phase in Session 1. In the observation phase, the top left window shows footage from the overview camera and the top right window shows footage from the observer camera.

**S3 Video. Example of the procedure for the control condition in Experiment 2.** The video shows an interaction with the generous partner (wearing a white spot-patterned poncho) and the selfish partner (wearing a dark camouflage print poncho) in the observation phase and the first trial of the test phase in Session 2. In the observation phase, the top left window shows footage from the overview camera and the top right window shows footage from the observer camera.

**S4 Video. Example of the procedure for the direct experience condition in Experiment 2.** The video shows the baseline, an interaction with the generous partner (wearing black) and the selfish partner (wearing white) in the observation phase, and the test phase in Session 1.

# Supplementary Figures and Tables

**S1 Table. Results of the full model for the string-pulling eavesdropping subset.**

| **Term** | **Estimate** | ***SE*** | **95% *CI*** | | ***z*** | ***p*** | **Min** | **Max** |
| --- | --- | --- | --- | --- | --- | --- | --- | --- |
|  |  |  | **Upper** | **Lower** |  |  |  |  |
| Intercept | -1.019 | 2.055 | 3.229 | -6.409 |  |  | -5.444 | -2.481 |
| Single trial in Session 1^a^ | -1.708 | 1.452 | 0.869 | -5.282 | -1.177 | .239 | -21.341 | -0.914 |
| First trial of Session 2^a^ | -3.097 | 1.520 | -0.544 | -6.964 | -2.038 | .042 | -23.924 | -2.499 |
| First trial of Session 2^b^ | -1.388 | 1.126 | 0.728 | 3.828 | -1.233 | .218 | -2.583 | -0.845 |
| Attention | 4.723 | 2.970 | 13.210 | -0.714 | 1.591 | .112 | 4.085 | 9.527 |

Estimate, standard error, confidence intervals, results of significance tests (Wald’s *z* approximation) and the range of estimates derived after excluding individuals one at a time.
^a^Baseline as reference level.
^b^Single trial in Session 1 as reference level.

**S2 Table. Results of the full model for the string-pulling reputation-learning subset.**

| **Term** | **Estimate** | ***SE*** | **95% *CI*** | | ***z*** | ***p*** | **Min** | **Max** |
| --- | --- | --- | --- | --- | --- | --- | --- | --- |
|  |  |  | **Upper** | **Lower** |  |  |  |  |
| Intercept | -1.476 | 2.047 | 2.133 | -6.878 |  |  | -2.723 | 1.575 |
| Z-transformed trial | -0.323 | 0.347 | 0.344 | -1.049 | -0.931 | .352 | -0.498 | -0.180 |
| Attention | 1.827 | 2.684 | 9.031 | -4.172 | 0.681 | .496 | -2.806 | 3.140 |

Estimate, standard error, confidence intervals, results of significance tests (Wald’s *z* approximation) and the range of estimates derived after excluding individuals one at a time.

**S3 Table. Results of the full model for the begging eavesdropping subset.**

| **Term** | **Estimate** | ***SE*** | **95% *CI*** | | ***z*** | ***p*** | **Min** | **Max** |
| --- | --- | --- | --- | --- | --- | --- | --- | --- |
|  |  |  | **Upper** | **Lower** |  |  |  |  |
| Intercept | 0.998 | 2.214 | 5.189 | -3.853 |  |  | -0.328 | 2.508 |
| Single trial in Session 1^a^ | 0.000 | 1.493 | 3.056 | -3.056 | 0.000 | 1.000 | -1.443 | 1.509 |
| First trial of Session 2 | -0.147 | 1.574 | 3.033 | -3.381 | -0.093 | .926 | -01.398 | 0.072 |
| Condition order: Experimental condition first^b^ | 0.031 | 1.679 | 3.465 | -3.582 | 0.019 | .985 | -0.809 | 1.129 |
| Condition: Experimental^c^ | -1.097 | 1.517 | 1.813 | -4.394 | -0.723 | .470 | -1.586 | 0.000 |
| Attention | -0.823 | 3.409 | 7.304 | -7.257 | -0.241 | .809 | -3.513 | 0.902 |
| Single trial in Session 1 × experimental condition first | -2.282 | 2.229 | 1.954 | -7.056 | -1.024 | .306 | -20.432 | -0.841 |
| First trial of Session 2 × experimental condition first | -2.183 | 2.313 | 2.260 | -7.075 | -0.944 | .345 | -17.280 | -1.084 |
| Single trial in Session 1 × experimental | 0.000 | 2.120 | 4.306 | -4.306 | 0.000 | 1.000 | -1.499 | 1.444 |
| First trial of Session 2 × experimental | 1.097 | 2.212 | 5.697 | -3.239 | 0.496 | .620 | -0.345 | 1.967 |
| Experimental condition first × experimental | 0.054 | 2.101 | 4.317 | -4.142 | 0.026 | .979 | -1.749 | 1.137 |
| Single trial of Session 1 × experimental condition first × experimental | 2.282 | 3.062 | 8.612 | -3.662 | 0.745 | .456 | 0.841 | 18.565 |
| First trial of Session 2 × experimental condition first × experimental | 2.200 | 3.118 | 8.614 | -3.883 | 0.706 | .480 | 0.308 | 17.740 |

Estimate, standard error, confidence intervals, results of significance tests (Wald’s *z* approximation) and the range of estimates derived after excluding individuals one at a time.
^a^Baseline as reference level.
^b^Condition order: control condition first as reference level.
^c^Condition: control condition as reference level.

**S4 Table. Results of the full model for the begging reputation-learning subset.**

| **Term** | **Estimate** | ***SE*** | **95% *CI*** | | ***z*** | ***p*** | **Min** | **Max** |
| --- | --- | --- | --- | --- | --- | --- | --- | --- |
|  |  |  | **Upper** | **Lower** |  |  |  |  |
| Intercept | -2.280 | 1.907 | 1.929 | -6.814 |  |  | -5.266 | 0.218 |
| Z-transformed trial | -0.239 | 0.595 | 0.966 | -1.550 | -0.401 | .688 | -0.971 | -0.005 |
| Condition order: Experimental condition first^a^ | 1.522 | 0.845 | 3.506 | -0.150 | 1.801 | .072 | 0.709 | 2.527 |
| Condition: Experimental^b^ | 1.218 | 0.803 | 2.915 | -0.323 | 1.517 | .129 | 0.996 | 1.720 |
| Attention | 1.314 | 2.951 | 8.125 | -5.537 | 0.194 | .656 | -1.976 | 6.301 |
| Z-transformed trial × experimental condition first | 0.240 | 0.736 | 1.847 | -1.275 | 0.326 | .745 | -0.082 | 0.973 |
| Z-transformed trial × experimental | 1.523 | 0.858 | 3.393 | -0.070 | 1.774 | .076 | 0.965 | 2.218 |
| Experimental condition first × experimental | -0.413 | 1.030 | 1.598 | -2.504 | -0.401 | .688 | -0.986 | 0.127 |
| Z-transformed trial × experimental condition first × experimental | -1.467 | 1.063 | 0.561 | -3.692 | -1.380 | .168 | -2.880 | -0.901 |

Estimate, standard error, confidence intervals, results of significance tests (Wald’s *z* approximation) and the range of estimates derived after excluding individuals one at a time.
^a^Condition order: control condition first as reference level.  ^b^Condition: control condition as reference level.

**S5 Table. Results of the full model for Session 1 of the direct experience subset.**

| **Term** | **Estimate** | ***SE*** | **95% *CI*** | | ***z*** | ***p*** | **Min** | **Max** |
| --- | --- | --- | --- | --- | --- | --- | --- | --- |
|  |  |  | **Upper** | **Lower** |  |  |  |  |
| Intercept | -10.377 | 4.751 | -3.127 | -73.551 |  |  | -11.164 | -9.878 |
| Trial^a^ | 19.832 | 7.471 | 83.747 | 4.591 | 2.654 | .008 | 18.716 | 20.606 |

Estimate, standard error, confidence intervals, results of significance tests (Wald’s *z* approximation) and the range of estimates derived after excluding individuals one at a time.
^a^Baseline as reference level.

**S6 Table. Results of the full model for Session 2 of the direct experience subset.**

| **Term** | **Estimate** | ***SE*** | **95% *CI*** | | ***z*** | ***p*** | **Min** | **Max** |
| --- | --- | --- | --- | --- | --- | --- | --- | --- |
|  |  |  | **Upper** | **Lower** |  |  |  |  |
| Intercept | 0.001 | 0.313 | 0.728 | -0.707 |  |  | -0.226 | 0.154 |
| Z-transformed trial | -0.084 | 0.269 | 0.515 | -0.717 | -0.312 | .755 | -0.240 | 0.095 |

Estimate, standard error, confidence intervals, results of significance tests (Wald’s *z* approximation) and the range of estimates derived after excluding individuals one at a time.
